# Autologous micro-fragmented adipose tissue for the treatment of diffuse degenerative knee osteoarthritis: an update at 3 year follow-up

**DOI:** 10.1186/s40634-018-0169-x

**Published:** 2018-12-19

**Authors:** A. Russo, D. Screpis, S. L. Di Donato, S. Bonetti, G. Piovan, C. Zorzi

**Affiliations:** 1 Orthopaedic Department, Sacro Cuore - Don Calabria Hospital, Negrar, VR Italy; 2 Orthopaedic Department, Humanitas Castelli Hospital, Via Mazzini 11, 24128 Bergamo, Italy

## Abstract

**Background:**

Conservative therapies for the treatment of knee degenerative processes are used before resorting to surgery; nonetheless, they may offer only short-term benefits. Encouraging preliminary results have been reported using mesenchymal stem cells (MSCs), either alone or in association with surgery. Among the many sources, adipose tissue has created a huge interest, because of its anti-inflammatory and regenerative properties ascribed to the cells of its stromal vascular fraction. We previously reported the safety and feasibility of autologous micro-fragmented adipose tissue as adjuvant for the surgical treatment of diffuse degenerative chondral lesions at 1 year. Here we present the outcomes of the same cohort of patients evaluated at 3 year follow-up. Micro-fragmented adipose tissue was obtained using a minimal manipulation technique in a closed system. The safety of the procedure was evaluated by recording type and incidence of any adverse event. The clinical outcomes were determined using the KOOS, IKDC-subjective, Tegner Lysholm Knee, and VAS pain scales taken pre-operatively and at 12 and 36 months follow-up.

**Findings:**

No adverse events, lipodystrophy cases at the harvesting site nor atypical inflammatory reactions at the joint level were reported. Of the 30 patients previously treated, one was lost, and seven received additional treatments in the period of observation. On average, the 22 patients that had no other treatments in the 3-year period showed that the results observed at 1 year were maintained. Moreover, 41, 55, 55 and 64% of the patients improved with respect to the 1-year follow-up in the Tegner Lysholm Knee, VAS, IKDC-subjective and total KOOS, respectively.

**Conclusion:**

Our results point to autologous and micro-fragmented adipose tissue injection as an innovative and safe approach for the management of diffuse degenerative knee chondropathy in the mid-term. The procedure is simple, affordable, minimally invasive, and compliant with the regulatory panorama.

## Background

The management of chondral disease is challenging because of its intrinsic poor healing potential. Biomechanical and biological changes may lead to the loss of tissue homoeostasis, resulting in an accelerated degeneration of the articular surface, eventually leading to end-stage osteoarthritis (OA).

Conservative therapies for the treatment of knee degenerative processes, such as non-pharmacological interventions, systemic drug treatment and intra-articular therapies are used before resorting to surgery; nonetheless, they may offer only short-term benefits. Encouraging preliminary results have been reported using mesenchymal stem cells (MSCs), either alone or in association with surgery. Among the many sources of MSCs, adipose tissue has created a huge interest in the context of cartilage regeneration (Pak et al. 2016; Ruetze and Richter 2014), due to its wide availability, ease to harvest and richness in mesenchymal cell elements within the so called stromal vascular fraction (De Girolamo et al. 2016; Caplan 2008; Caplan and Correa 2011; Caplan and Dennis 2006). Moreover, MSCs from adipose tissue are characterized by marked anti-inflammatory and regenerative properties, which make them an excellent tool for regenerative medicine purposes (De Girolamo et al. 2016; Caplan 2008; Caplan and Correa 2011; Caplan and Dennis 2006). Nevertheless, preparation of autologous MSCs for injection requires ex vivo culture from a good manufacturing practice facility, which makes the process laborious and expensive (Ährlund-Richter et al. 2009; Arcidiacono et al. 2012; Sensebé et al. 2010). An increasing number of adipose tissue-derived cell isolation systems, allowing for minimal manipulation, have been developed in the last years. We previously reported the safety and feasibility of autologous micro-fragmented adipose tissue as adjuvant for the surgical treatment of diffuse degenerative chondral lesions at 1 year follow-up (Russo et al. 2017). Here we present the outcomes of the same cohort of patients evaluated at 3 year follow-up.

## Methods

The original study was approved by the Ethics Committee of Verona and Rovigo - Italy (protocol n° 10,227, March 1st, 2016). An extension of the study protocol has been conceded by the same authority to evaluate the results at 3 years (protocol n° 14,505, March 14th 2018) and written informed consent was obtained from all patients.

Study design and population, surgical techniques, post-op rehabilitation protocol, safety and clinical evaluation were previously described (Russo et al. 2017). Briefly, 30 patients, affected by diffuse degenerative chondral lesions of different degrees of severity, were treated with autologous and micro-fragmented adipose tissue between 1^st^ January 2014 and 31^st^ December 2014. Of these 30 patients, 24 (80%) also had an associated surgery (ACL/LCL reconstruction, high tibial osteotomy, meniscectomy), while six (20%) underwent arthroscopy alone. For the 3 year follow-up all the patients were re-contacted and clinically evaluated by the same clinicians.

## Findings

Of the 30 patients treated with autologous micro-fragmented adipose tissue, eight also had meniscal surgery, five plate removal, three osteotomy, two ligament surgery, two microfractures, and four other surgical procedures. The remaining six had arthroscopy alone. Despite the heterogeneity of the associated surgical procedures all the patients shared the presence of chondral lesions of different degrees of severity (Russo et al. 2017).

At 3 years follow-up, one patient was lost, and seven (23%) received additional treatments in the period of observation, and therefore have been considered failures. In detail, between 18 and 30 months, one patient had three injections of hyaluronic acid and the other six had multiple injections of platelet rich plasma. Background data on this subpopulation is reported in Table 1.

**Table 1:** Background data of the failures (*n* = 7)

Age y/o	
Mean	36.3
Standard deviation	7.3
Type chondropathy	
FC	4 (57%)
TP	2 (29%)
PF	6 (86%)
Three-compartment	2 (29%)
Associated surgery	
YES	5 (71%)
NO	2 (29%)

*FC* femoral condyle, *TP* tibial plateau, *PF* patellofemoral

No adverse events, lipodystrophy cases at the harvesting site nor atypical inflammatory reactions at the joint level were reported in the 3 year period for all the 29 patients.

On average, the 22 patients that had no other treatments in the 3 year period (Table 2) showed that the results observed at 1 year were maintained (T36 vs. T12, *p* > 0.05). Moreover, 41, 55, 55 and 64% of the patients improved with respect to the 1-year follow-up in the Tegner Lysholm Knee, VAS, IKDC-subjective and total KOOS, respectively.

**Table 2:** Background data of the population (*n* = 22)

Age y/o	
Mean	44.7
Standard deviation	11.4
Gender	
M	14 (64%)
F	8 (36%)
BMI	
Mean	25.9
SD	3.3
Sport	
Professionals	1 (4%)
Amateurs	9 (41%)
Occasional	5 (23%)
Inactive	7 (32%)
Grade chondropathy (ICRS classification)	
II	7 (32%)
III	6 (27%)
IV	9 (41%)
Type chondropathy	
FC	17 (77%)
TP	14 (64%)
PF	14 (64%)
Three-compartment	9 (41%)
Associated surgery	
Yes	18 (82%)
No	4 (18%)

*FC* femoral condyle, *TP* tibial plateau, *PF* patellofemoral

**Fig. 1:**
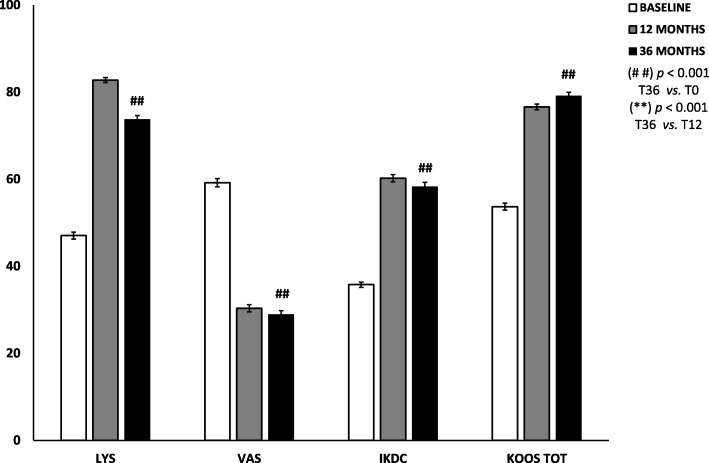
Trend of functional improvements of Tegner Lysholm knee, VAS pain, IKDC subjective and total KOOS pre-operatively (white bars), at 12 (grey bars) and 36 months (black bars) after micro-fragmented adipose tissue injection. Results are expressed as mean and standard error

Compared to pre-operative values, more than 50% of the patients improved at least 20 points in all the considered scores, and, surprisingly, 55% of the patients improved at least 30 points in the VAS pain scale. A summary of the results is reported in Fig. 1.

## Discussion

The main finding of this study is that the beneficial effect of autologous micro-fragmented adipose tissue as adjuvant for the treatment of diffuse degenerative chondral lesions is maintained in the mid-term. In addition, no complications were observed in the 3 year period showing the safety profile of this procedure. No patient, including the seven patients who received additional treatments, worsened compared to the pre-operative condition.

Despite the heterogeneity of the associated surgical procedures all the patients shared the presence of chondral lesions of different degrees of severity, which may have been responsible for the impairment in function and pain.

As reported in literature, articular surface damages, especially when diffused (three compartment OA), positively correlate with a decay in the outcomes in patients who received knee surgery for other reasons (Bonasia et al. 2014; Røtterud et al. 2012; Saithna et al. 2014; Su et al. 2018; Verdonk et al. 2016). Published data shows a decline in the clinical results in the mid to long-term for arthroscopic and chondral debridement procedures in cases of initial knee OA (Su et al. 2018). Some authors assessed the effectiveness of the arthroscopic or conservative treatments in patients diagnosed with knee OA (Kellgren-Lawrence grade 2 to 4) with 5 years of follow-up, concluding that arthroscopy provided no benefit in decreasing or delaying arthroplasty and that it can relieve symptoms only up to 2 years (Su et al. 2018). The same observation has been reported for ligament reconstruction, where the short and mid to long-term benefits are inferior in patients who have cartilage lesions. In a study of a cohort of ACL-injured patients with full-thickness cartilage lesions (ICRS grade III–IV), the authors showed that ACL-injured patients with full-thickness cartilage lesions reported worse outcomes and minor improvement after ACL reconstruction compared to patients without cartilage lesions at 2–5 years follow-up, although no significant differences between the two groups at the time of ACL reconstruction were present. This means that the observed differences between the groups must have occurred during the follow-up period (Røtterud et al. 2012). Furthermore, the outcomes of osteotomy procedures in patients with diffuse degenerative knee chondropathy worsen in the mid to long-term (Bonasia et al. 2014; Saithna et al. 2014). In a study reporting the results of a case series of opening wedge distal femoral varus osteotomies for valgus lateral knee OA, it is shown that re-operation for non-arthroplasty related surgery was common due, besides others, to infection and persistence of symptoms (Saithna et al. 2014). With regard to meniscectomy, in a recently published paper it is concluded that meniscus therapy including partial meniscectomy, meniscus suture, and meniscus replacement has proven beneficial effects in long-term studies in patients without cartilage damage, supporting the hypothesis that meniscectomy increases the risk of cartilage degeneration (Verdonk et al. 2016).

Based on the aforementioned published evidences, we should have expected, in the mid-term, a decay of the outcomes. Notably, the results have been maintained with no significant differences in all the evaluated parameters with respect to the 1 year follow-up assessment. Furthermore, in line with that already observed at 1 year, the patients with lesions in more than one compartment had higher and statistically significant improvements compared to patients with lesions in only one compartment (*p* < 0.01). This finding supports our hypothesis of using micro-fragmented adipose tissue for the treatment of the diffuse degenerative knee pathology as an adjuvant of the surgical procedures. Indeed, the maintenance of stable results at the last follow-up leads to hypothesize a protective role of micro-fragmented adipose tissue in a further chondral degeneration.

The seven patients who received additional biological therapies in the 3-year period, were young (mean age 36.3 ± 7.3 vs. 44.7 ± 11.4), very active in sport and 6 out of 7 had a patellofemoral chondropathy. Their conditions after 1 year did not worsen, but they probably needed an additional biological treatment because of their high functional demands and the presence of the patellofemoral chondropathy, which is a negative prognostic element, even if the small number of patients does not allow for any statistical correlation.

## Conclusion

Our results point to autologous and micro-fragmented adipose tissue injection as an innovative and safe approach for the management of diffuse degenerative knee chondropathy in the mid-term. The procedure is simple, affordable, minimally invasive, and compliant with the regulatory panorama. Despite it would be advisable to make a comparison with the same associated surgery alone, based on our clinical experience and literature data we certainly recommend the use of micro-fragmented adipose tissue as an adjuvant to the surgical procedure where chondral degeneration is present.

## Abbreviations

ASCs: Adipose-derived stem cells
FC: Femoral condyle
IKDC: International knee documentation committee
KOOS: Knee injury and osteoarthritis outcome score
MSCs: Mesenchymal stem cells
PF: Patellofemoral
TP: Tibial plateau
VAS: Visual analogue scale
